# Osteo-Porosis, Not Millet Disease

**Published:** 1894-01

**Authors:** W. H. Mattsen

**Affiliations:** Camp Ground, Penn.


### OSTEO-POROSIS, ***NOT*** MILLET DISEASE.

By W. H. Mattsen, V.D.M.

In the November number of the Journal there is an article entitled "Millet Disease," by T. D. Hinebauch, M.S., V.S. I must certainly take objection to his diagnosis of the cases he gives in point. The symptoms, as he gives them, point conclusively to osteo-porosis. He speaks of the great soreness of the muscles of back, chest and loins, of haunches being very sore and painful to the touch ; of course, when pressure is put upon these muscles, in osteo-porosis there is more or less strain on their bony attachments, and these cases, having the disease in an acute form, there would be more pain evinced upon pressure than we generally see. He speaks of the high temperature in some cases ; at the outset it might be due to the severe pain alone, *most likely it was.* The increased action of kidneys at first and diminished action afterwards might be the result of pain due to general diseased condition of the bones, or it might be an effort of nature to relieve the system of some poisonous material. The bowels, he says, are constipated, any fever will tend to that condition; anyway there is some lack of nutrition in the animal. The cramped position is due more to the pain in the joints and body of the bones than in the muscles. The disposition to keep still, the pain the animal showed when moved, are characteristic symptoms of osteo-porosis. The inability to get up when down, also the pain in the joints is severe.

The pleurodynia he speaks of, is most likely the pain caused by the ribs and their articulations being so very sore. The pleurisy must be a mistake. He says, these severe symptoms usually subside in from twenty-four to forty-eight hours with vigorous treatment, *then* the joints become affected and *typical* form is established. Now, then, he is coming more to the point and to our conclusions.

These symptoms which he gives show the disease in an acute form, the increased temperature, the increased activity of kidneys, the great pain, *seemingly*, of the muscles, and the lameness of the animal, all show that the disease was very rapid and severe. Usually we see it in a slower form, and find no rise in temperature, in fact, no severe symptoms, but lameness and soreness of joints and often soreness of the spinous processes, probably these acute symptoms he found were due to the surroundings of the animals, as he says, their stables were very damp, in fact, the ceiling covered with moisture and dropping on the horses; and he also says that *young* horses have it in a milder form than older ones.

We now know that young horses *can be* cured of osteo-porosis if the diagnosis is made early enough and the proper treatment is used; but for older ones, I doubt if anything can be done for them. He gives three cases as being an average of them all, the first, he says, recoved in about ten days; now she *might have had* azoturia and gotten over it all right; her symptoms do not coincide with next case.

Case II. Great lameness in left hind leg, all the joints very sore and tender to the touch, would stand by awhile, when down would not rise, was placed in slings and in her struggles ruptured the perforatus tendon of the right hind foot; *just that lesion shows her trouble.*

Case III. Symptoms about the same as Case II; in their attempt to raise her with slings the gastrocnemius was ruptured from the os calcis. *Proof No. 2.*

*Post-mortem Lesions.*—The rupture of the different muscles from their attachments, the fact that in some cases pieces of bone were torn loose, the diminished supply of synovial fluid, the indentations on the ends of the bones and articular surfaces, the wearing away of the articular cartilages and the bones becoming spongy, show perfectly, plainly, that his "Millet Disease" is osteo-porosis and nothing else. After he found these lesions on post-mortem, *he surely ought to have recognized the trouble.*

He says: "Most of the cases occurred in poorly ventilated barns." What has ventilatiou got to do with the result of millet as feed to horses? I would like him to tell us.

The experiments he made with millet as a feed seem to me a waste of time, as the result he gives shows nothing whatever. One horse had colic twice within a week, and the other showed a queer gait for a little time. I have fed millet to my own horses for weeks together, and have known horses all around me to have been fed on it, with no hay at all, and I have never yet seen any bad effects from it. It seems to me, if it did cause trouble, it would simply be some boned trouble or colic.

CAMP GROUND, PENN.

